# Epicosm—a framework for linking online social media in epidemiological cohorts

**DOI:** 10.1093/ije/dyad020

**Published:** 2023-02-27

**Authors:** Alastair R Tanner, Nina H Di Cara, Valerio Maggio, Richard Thomas, Andy Boyd, Luke Sloan, Tarek Al Baghal, John Macleod, Claire M A Haworth, Oliver S P Davis

**Affiliations:** Medical Research Council Integrative Epidemiology Unit, University of Bristol, Bristol, UK; Medical Research Council Integrative Epidemiology Unit, University of Bristol, Bristol, UK; Department of Population Health Sciences, Bristol Medical School, University of Bristol, Bristol, UK; Medical Research Council Integrative Epidemiology Unit, University of Bristol, Bristol, UK; Department of Population Health Sciences, Bristol Medical School, University of Bristol, Bristol, UK; Department of Population Health Sciences, Bristol Medical School, University of Bristol, Bristol, UK; Department of Population Health Sciences, Bristol Medical School, University of Bristol, Bristol, UK; School of Social Sciences, Cardiff University, Cardiff, UK; Institute for Social and Economic Research, University of Essex, Colchester, UK; Department of Population Health Sciences, Bristol Medical School, University of Bristol, Bristol, UK; School of Psychological Science, University of Bristol, Bristol, UK; Alan Turing Institute, British Library, London, UK; Medical Research Council Integrative Epidemiology Unit, University of Bristol, Bristol, UK; Department of Population Health Sciences, Bristol Medical School, University of Bristol, Bristol, UK; Alan Turing Institute, British Library, London, UK

**Keywords:** Social media, epidemiology, cohort studies, longitudinal studies, data science, Big Data, mental health, wellbeing, data linkage, ALSPAC

## Abstract

**Motivation:**

Social media represent an unrivalled opportunity for epidemiological cohorts to collect large amounts of high-resolution time course data on mental health. Equally, the high-quality data held by epidemiological cohorts could greatly benefit social media research as a source of ground truth for validating digital phenotyping algorithms. However, there is currently a lack of software for doing this in a secure and acceptable manner. We worked with cohort leaders and participants to co-design an open-source, robust and expandable software framework for gathering social media data in epidemiological cohorts.

**Implementation:**

Epicosm is implemented as a Python framework that is straightforward to deploy and run inside a cohort’s data safe haven.

**General features:**

The software regularly gathers Tweets from a list of accounts and stores them in a database for linking to existing cohort data.

**Availability:**

This open-source software is freely available at [https://dynamicgenetics.github.io/Epicosm/].

## Introduction

Digital footprint data, such as data from social media, banking and shopping, online searches, and apps such as exercise trackers, offer huge potential for epidemiological studies to derive new digital phenotypes based on real human behaviour. For example, research predicting mental health from digital data has been increasing since 2013,^1^ and progress in this area could improve access to mental health care, such as by offering overstretched services a way of supporting patients between check-in occasions. Early detection of problems through digital phenotyping could lead to early interventions that prevent the development of more complex issues. This might work at both an individual level^2^ and a strategic level, where services can be put in place to meet anticipated demands of populations such as student groups,^3^^,^^4^ emergency workers^5^ or geographical regions,^6^ an approach that proved particularly valuable during the COVID-19 pandemic.^7–10^ Research in this field has developed methods for inferring a wide range of outcomes, including social anxiety,^11^ suicidality,^12^^,^^13^ depression,^14–16^ wellbeing^6^^,^^17^ and happiness.^18^^,^^19^ This makes social media data a potentially valuable source of information for epidemiological studies, such as birth cohorts, to supplement more traditional approaches. Inference from social media data has the potential to provide high temporal resolution data on mental wellbeing on daily or even hourly timescales, and research using these data could advance our understanding of mental health time courses, aid early diagnosis and inform public health interventions and policies,^20^^,^^21^ opportunities recognized by funding bodies, such as United Kingdom Research and Innovation (UKRI) and the Wellcome Trust, in their cohort data linkage strategies.^22^^,^^23^

Conversely, researchers developing approaches for inferring phenotypes from these novel data can benefit from the resources provided by epidemiological cohorts. Historically, studies using social media have rarely had good knowledge of the samples they were studying, risking demographic bias and unmeasured confounding. Similarly, these studies have rarely had access to good ‘ground truth’ measures of their phenotypes of interest. Epidemiological cohorts, with their well characterized participants and state-of-the-art phenotyping, offer an opportunity for a step change in the quality of research in this area by allowing straightforward validation of new digital measures against gold-standard, symptom-based assessments and diagnoses in a known population.

Despite these advantages, currently few longitudinal cohorts have linked digital footprint data, because of the specific challenges. For example, social media data are difficult to anonymize; with a publicly available platform such as Twitter, there is no way for a cohort to share user names or Tweets without identifying cohort participants. This is particularly important because cohorts rely on a long-term trust relationship with participants, and the disclosure of personal data could lead to reputational damage for the cohort and a decrease in participation. Such challenges mean that cohorts could benefit greatly from software designed in collaboration with cohort leaders and participants to meet their specific needs. With this in mind, we worked closely with stakeholders from the Avon Longitudinal Study of Parents and Children (ALSPAC) and other CLOSER (based at the University College London Social Research Institute) cohorts to design the Epicosm software to address these special requirements.

Although social media harvesting software products are widely available, most require significant programming skills run in a way useful to longitudinal cohorts, such as collecting new data regularly from a list of specific users. Similarly, most social media harvesters are not well documented, and do not provide functions such as data management or built-in approaches for inferring common digital phenotypes from datasets. In contrast, Epicosm is designed to be relatively straightforward to set up and run on servers in the heterogeneous computing environments inside cohorts’ data safe havens, allowing long-term linking of social media time lines from a list of users, storage of information in a flexible database structure, and automated and modular processing of the data using several widely used coding algorithms. At the time of writing, Epicosm’s focus is on harvesting data from Twitter. However, the software has been designed following software engineering best practices, including modular organization to allow expansion to other social media platforms, Open Source code available on GitHub, and documentation written with future collaborators and maintainers in mind. The data collected by Epicosm form the basis for a depersonalized dataset of information, derived from social media, which can be shared with researchers through a cohort’s usual data access mechanisms. As social media big data continue to evolve, Epicosm provides robust data acquisition tools so that epidemiology can benefit from these rich sources of information about the daily lives and behaviours of people and populations.

## Implementation

Epicosm is an open-source project freely available under a GPL version 3 licence from GitHub [dynamicgenetics.github.io/Epicosm/], along with full documentation. Collaborators are welcome to branch, fork or issue pull requests [for example, updating in response to changing API (Application Programming Interface) authentication] and to add custom functionality. The modular nature of the software suite allows adaptation for alternative platforms, allowing any typical API response to be archived in a local database for later analysis. Together, the software engineering principles applied in its development promote collaboration to maintain and expand Epicosm’s scope, and allow it to act as a foundation for a variety of research.

For data management, Epicosm uses the open-source, non-relational document database MongoDB [mongodb.com]. MongoDB was chosen for flexibility: the schema is consistent with Java Script Object Notation (JSON) data structure, a common format for API responses. This allows the storage of a variety of types of data (from plain text with metadata to images and other media), and accommodates adaptation of Epicosm to variation in API responses over time and across a range of social media platforms.

We anticipate that Epicosm will be installed and managed by cohort data managers: these staff typically have permissions to process identifiable participant data and are responsible for the post-processing (for example de-personalization) needed prior to sharing with researchers. In development we have been sensitive to user requirements, keeping requisite skills marginal: some basic experience of the command line interface is expected, but no programming experience is required and we provide full instructions for setting up and running the software. The repository also contains links to resources to support new users.

The steps to gather information from the Twitter API using Epicosm are as follows. The user must provide two files: (i) a list of participants’ Twitter user names (also known as ‘screen names’ or ‘handles’); and (ii) a Twitter API bearer token to authorize API requests. Once these are in place, Epicosm is ready to run and carries out the following processes.

- Credentials are verified by Twitter’s API.
- The API converts screen names to unique and persistent identification (ID) numbers (this enables the tracking of participant accounts longitudinally, even where participants change screen names).
- Epicosm then requests Twitter timelines—that is, the user’s tweets (posts by the user) and re-tweets (re-posts of other users’ posts)—from each ID number. With an authorised academic research account, the complete tweet history of each user is available.
- Finally, each record (a single JSON document for each tweet) is stored in MongoDB. The tweet harvest can be scheduled to repeat at regular intervals specified by the user.

Various options are available, depending on the specific consent obtained from participants, including acquiring the list of ‘followed’, third-party Twitter account names. Public followed accounts can also be harvested for their tweets: the content of this harvest approximates the ‘feed’ that a user is presented with by Twitter (or at least, the pool of tweets available for Twitter to present to the user). In contrast to the original user tweet harvest, this harvest will only acquire posts made in the last 7 days (but can be repeated weekly). A full-archive harvest of followed accounts is theoretically possible, but not currently implemented in Epicosm: users can each follow thousands of accounts, each of which may have a large history of tweets, especially if they are intensely managed (for example, celebrity accounts) or automated accounts (for example, sports results or the weather).

Epicosm includes a selection of widely used algorithms (Box 1) for deriving sentiment (and other) information from Twitter data: LabMT,^18^ VADER,^24^ LIWC2015^25^ and TextBlob^26^ (note that, for licensing reasons, LIWC analysis requires the users to acquire a dictionary from the LIWC developers). Epicosm applies the analyses to each tweet, and appends these to each record in labelled database fields. The software provides implementations of these commonly used measures, as a demonstration of how phenotypes can be automatically added to the data base and because they are likely to be requested by researchers. However, the platform is flexible to allow users to derive novel phenotypes through the addition of custom algorithms or new dictionaries to allow analysis of languages other than English, and we anticipate that cohorts will employ a variety of their own approaches to derive information from the Twitter data once Epicosm has downloaded and stored it.

Box 1 Sentiment analysis methods

Sentiment analysis, the most common approach applied to derive information from social media data, is the inference of emotions, opinions and attitudes from written text. Output metrics vary depending on the methodology, but common inferences include positive or negative emotions or a composite of both [for example, VAD ER^29^ (Valence Aware Dictionary and sEntiment Reasoner)]. Some methods also aim to derive more specific emotional and syntactic content, for example LIWC^30^ (Linguistic Inquiry and Word Count) infers over 70 categories from emotions aor gender specificities to politics or food.

A commonly used methodology is the ‘dictionary approach’. Individual words are first assigned sentiment scores by a group of participants, to build up a dictionary. For example, words such as ‘death’, ‘hate’ and ‘hell’ might be assigned negative scores, and ‘friend’, ‘happy’ and ‘love’ are generally rated more positively. The text is then assigned a mean score based on the dictionary words it contains (or a relative frequency for categorical dictionaries). This straightforward approach is limited by features of natural language such as negation, neologism, irony or sarcasm, but these are often equally difficult for human readers to understand, and their influence can be mitigated by applying more sophisticated natural language processing and machine learning approaches that aim to interpret sentence structure or do not assume the direct correspondence between the dictionary definition of words and the associated phenotypes. Linking social media data in epidemiological cohorts provides a crucial tool to develop these new approaches, by providing access to linked independent outcome (‘ground truth’) measures and demographic information about the populations studied.

## Use

As an approximate guide based on a random sample, 1000 users typically have an acquirable history of around 700 000 tweets, leading to a database size of around 3.5 GB, although the software will also allow data collection from much larger samples, limited only by storage space and the Twitter API’s rate limits. When first run, Epicosm will attempt to gather the full tweet time line history for each user. Subsequent harvesting operations will return only the tweets more recent than the latest tweet already in the database. At the time of writing, data can be acquired at about a million tweets per hour, but this will be highly dependent on connection speed, network activity and any rate-limiting measures Twitter impose (i.e. where they restrict the rate of download via the API).

As an example of expected use, we gathered tweets from a list of around 800 Twitter accounts of consenting participants in ALSPAC, an epidemiological birth cohort of around 15 000 families recruited between 1991 and 1992 in the historical county of Avon in the west of the UK.^27^ ALSPAC was interested in understanding the potential of these novel data to infer changes in mental health over time. Participants provided informed consent and ethical approval was provided by the ALSPAC Ethics and Law committee. We used Epicosm to link Twitter data as proof-of-principle. Of course, different populations use social media platforms in different ways, and this evolves with time. Twitter users, in the UK at least, are on average younger and slightly more likely to be male than the general population,^28^ although there is less age bias than previously assumed, with good representation from all age groups. For the ALSPAC young people at 24 years old, there was little difference in Twitter use across gender, ethnicity and parental employment groups, although those who had completed Advanced Level qualifications (post-16 school leaving examinations) were slightly more likely to use Twitter (58% compared with 51%).^29^ Despite the potential for bias in the sample, cohorts are the ideal for collecting this type of data because the biases are often identifiable. Twitter is currently among the social media platforms most open to academic research, but our intention is to expand Epicosm’s capabilities in future to include linking other forms of social media, subject to API restrictions.

The Twitter data linkage in ALSPAC was guided by conversations with cohort participants to understand the acceptability of this use of data and to establish appropriate safeguards,^30^ and with cohort data managers and linkage experts to understand the requirements for running the software and retrieving the data. These insights emphasize the wider evidence from participants^30^^,^^31^ that it is a necessity for data accessed by researchers to be de-personalized, and that study data managers operate in a trusted role where they are able to capture identifiable data and process these so they are suitable for dissemination to researchers.

We developed a data management protocol that ensured that Twitter linkage fitted with ALSPAC’s linkage data pipeline model, acquiring tweets from consenting participants via the Twitter Application Programming Interface (API) and depositing these in raw form in a permanent, versioned MongoDB data base. Data bases such as MongoDB are particularly useful for social media data because they store data in the form of documents that are very similar to the responses received from social media APIs. In this case, each document corresponded to a tweet and its associated metadata. The ALSPAC data managers (who have exclusive access to participant identifiers) followed a protocol that involved:

- the implementation of consent and withdrawal;
- providing the software with a list of Twitter user names which guided the collection of data from the Twitter API;
- subsequent curation of the data in the MongoDB database (including documentation and versioning);
- the management of participant identifiers to enable linkage to other cohort data;
- ensuring that the raw captured data were sufficiently depersonalized to share as structured data outputs, while retaining full Twitter content within the cohort’s data safe haven for the duration of the study so that it could be repurposed for future research needs.

## Conclusion

Social media data offer huge potential for digital phenotyping in epidemiological cohort studies to complement traditional measures. Equally, epidemiological cohorts have much to offer digital footprint researchers. We have described the software Epicosm and how it can be used by epidemiologists to expand existing cohort datasets. The software provides a robust foundation for Twitter data acquisition, and enables the exploration of participants’ digital footprints to address important health and social research questions. As the importance of online community and communication increases (especially in light of global health events such as the COVID-19 pandemic), Epicosm offers epidemiologists a practical way to expand their work into novel types of data and methodologies, and opens up the valuable data already held by longitudinal population cohorts to new research communities.

## Ethics approval

This study and all related work were approved by the ALSPAC Ethics and Law Committee (Haworth.Davis.B2934).

## Data Availability

This Open Source software is freely available at [https://dynamicgenetics.github.io/Epicosm/].
